# Supplementary material for: Nuclear Argonaute protein NRDE-3 switches small RNA partners during embryogenesis to mediate temporal-specific gene regulatory activity
Source: eLife. 2025 Mar 13;13:RP102226. doi: 10.7554/eLife.102226 (PMC11906161; doi:10.7554/eLife.102226)
Supplement: Figure 1—figure supplement 2—source data 1. [file elife-102226-fig1-figsupp2-data1.zip › Figure 1-Figure Supplement 2-Source Data 1.pdf]

Figure 1—figure supplement 2D

Gel1-exposure 25.5s

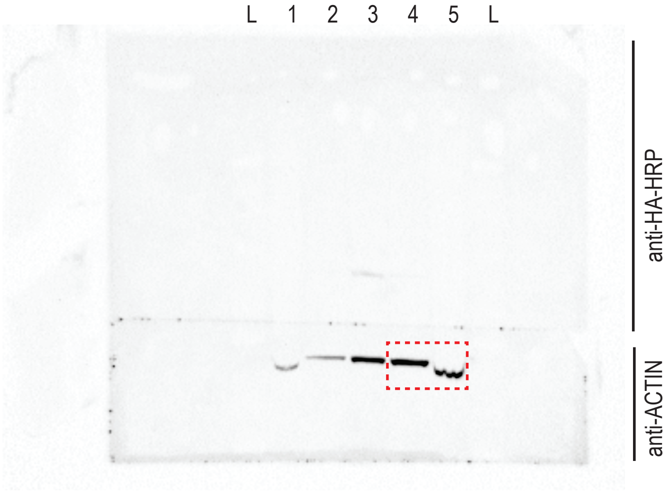

Gel2- exposure 517.9s

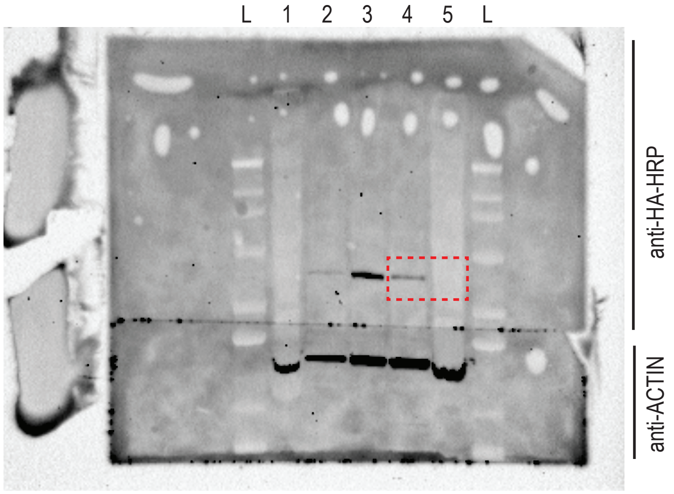

L: ladder
lane1: wild-type N2 (young adult-200 worms)
lane2: ENRI-1::mCherry::2xHA (mixed stage)
lane3: ENRI-1::mCherry::2xHA (mixed stage)
lane4: ENRI-1::mCherry::2xHA (young adult-200 worms)
lane5: wild-type N2 (young adult-200 worms)
