# Supplementary figures and images for: Nuclear Argonaute protein NRDE-3 switches small RNA partners during embryogenesis to mediate temporal-specific gene regulatory activity
Source: eLife. 2025 Mar 13;13:RP102226. doi: 10.7554/eLife.102226 (PMC11906161; doi:10.7554/eLife.102226)

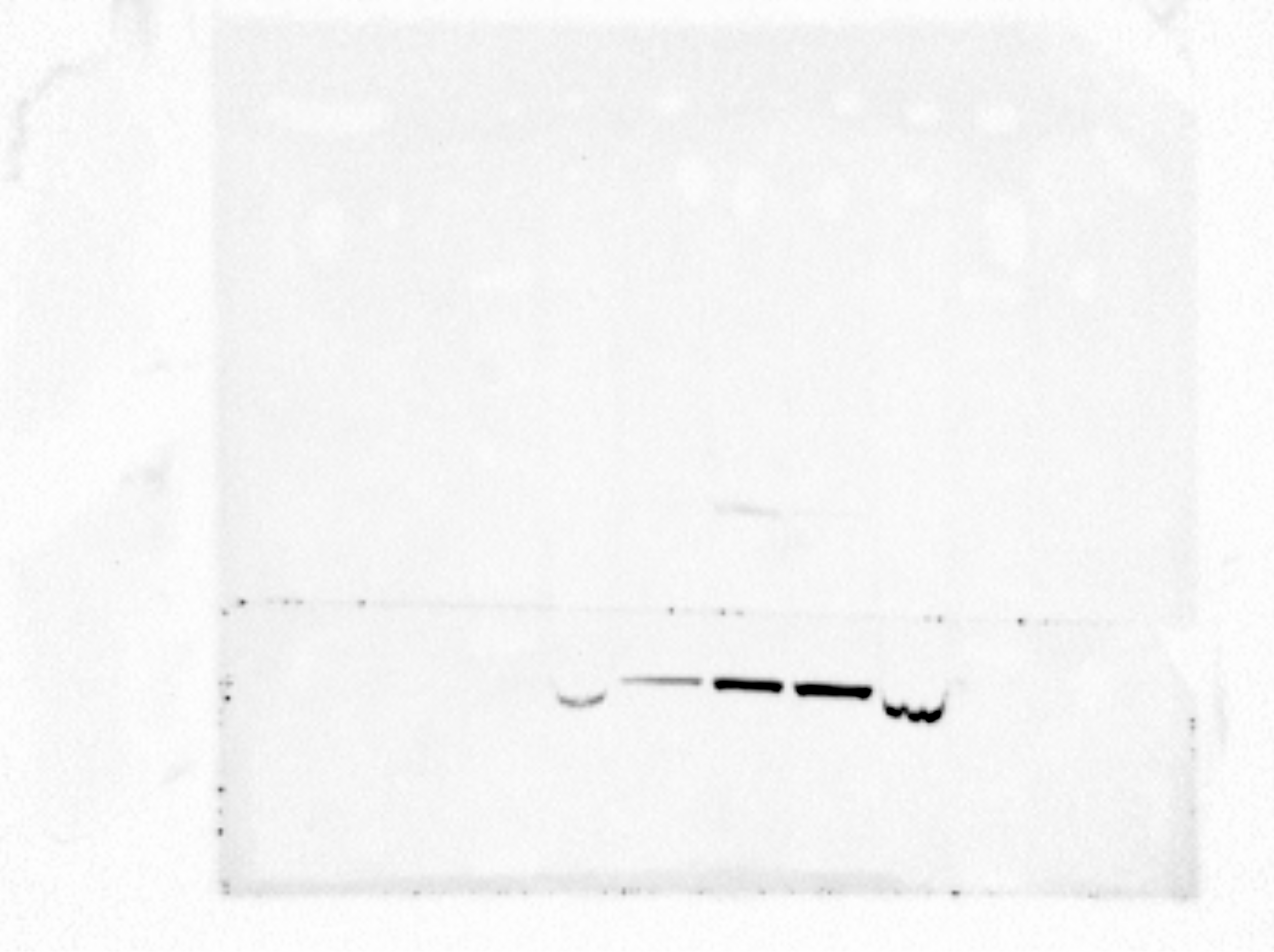

Supplement: Figure 1—figure supplement 2—source data 2. [file elife-102226-fig1-figsupp2-data2.zip › Figure 1-Figure Supplement 2-Source Data 2/Figure 1-Figure Supplement 2-Source Data 1-blot 1.tif]

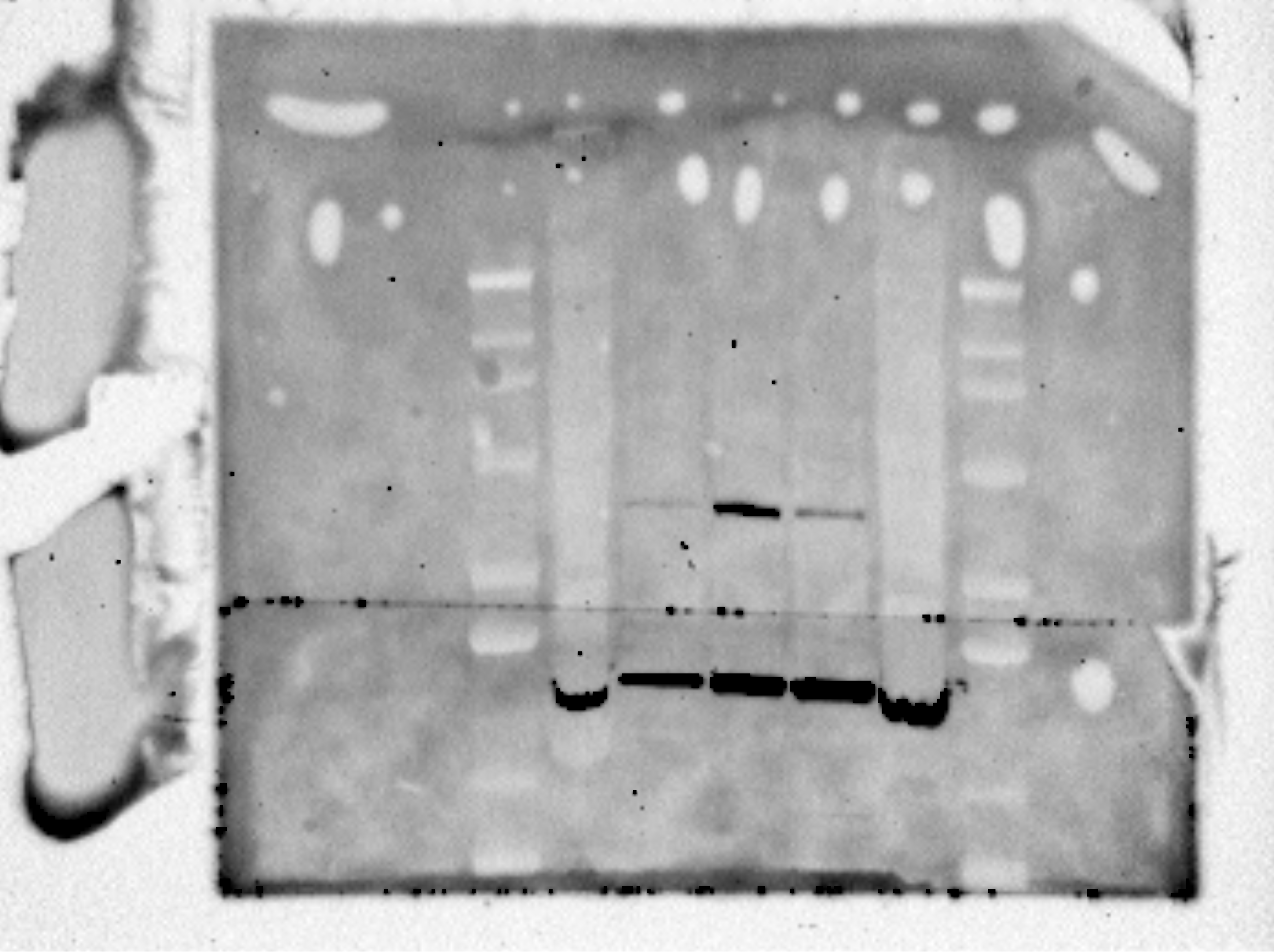

Supplement: Figure 1—figure supplement 2—source data 2. [file elife-102226-fig1-figsupp2-data2.zip › Figure 1-Figure Supplement 2-Source Data 2/Figure 1-Figure Supplement 2-Source Data 1-blot 2.tif]

Figure 2 -- figure supplementary 1B

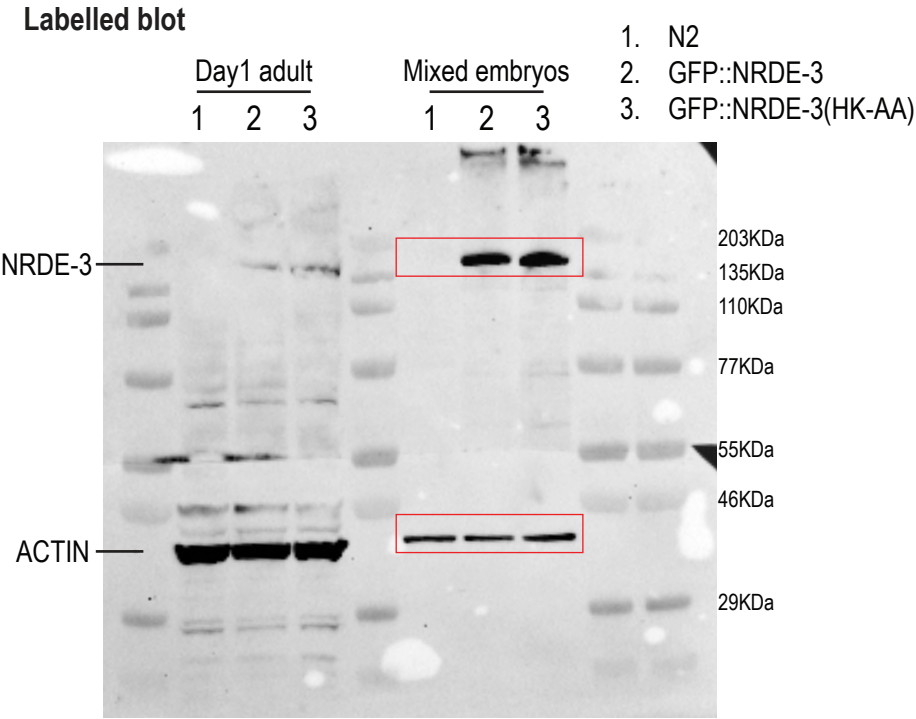

Supplement: Figure 2—figure supplement 1—source data 1. [file elife-102226-fig2-figsupp1-data1.zip › Figure 2-Figure Supplement 1-Source Data 1.pdf]

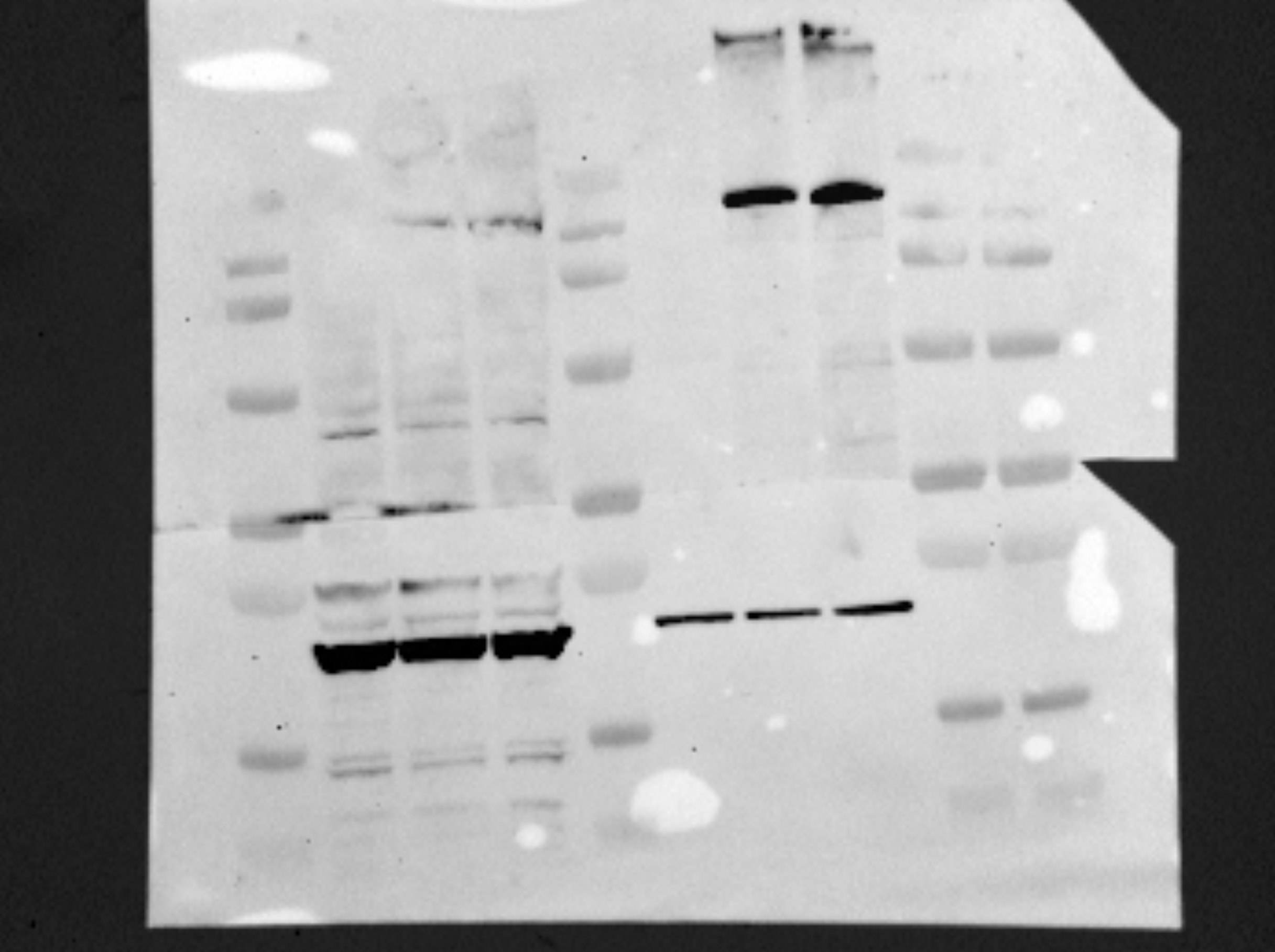

Supplement: Figure 2—figure supplement 1—source data 2. [file elife-102226-fig2-figsupp1-data2.zip › Figure 2-Figure Supplement 1-Source Data 2/Figure 2-Figure Supplement 1-Source Data 1-blot 1.tif]

Figure 3—figure supplement 1A

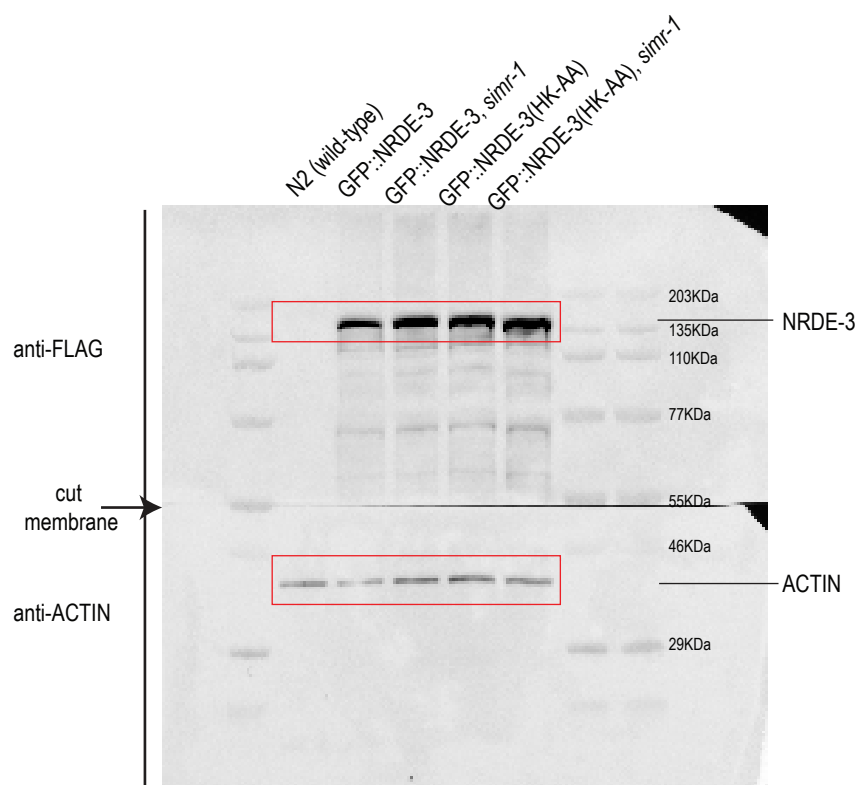

Supplement: Figure 3—figure supplement 1—source data 1. [file elife-102226-fig3-figsupp1-data1.zip › Figure 3-Figure Supplement 1-Source Data 1.pdf]

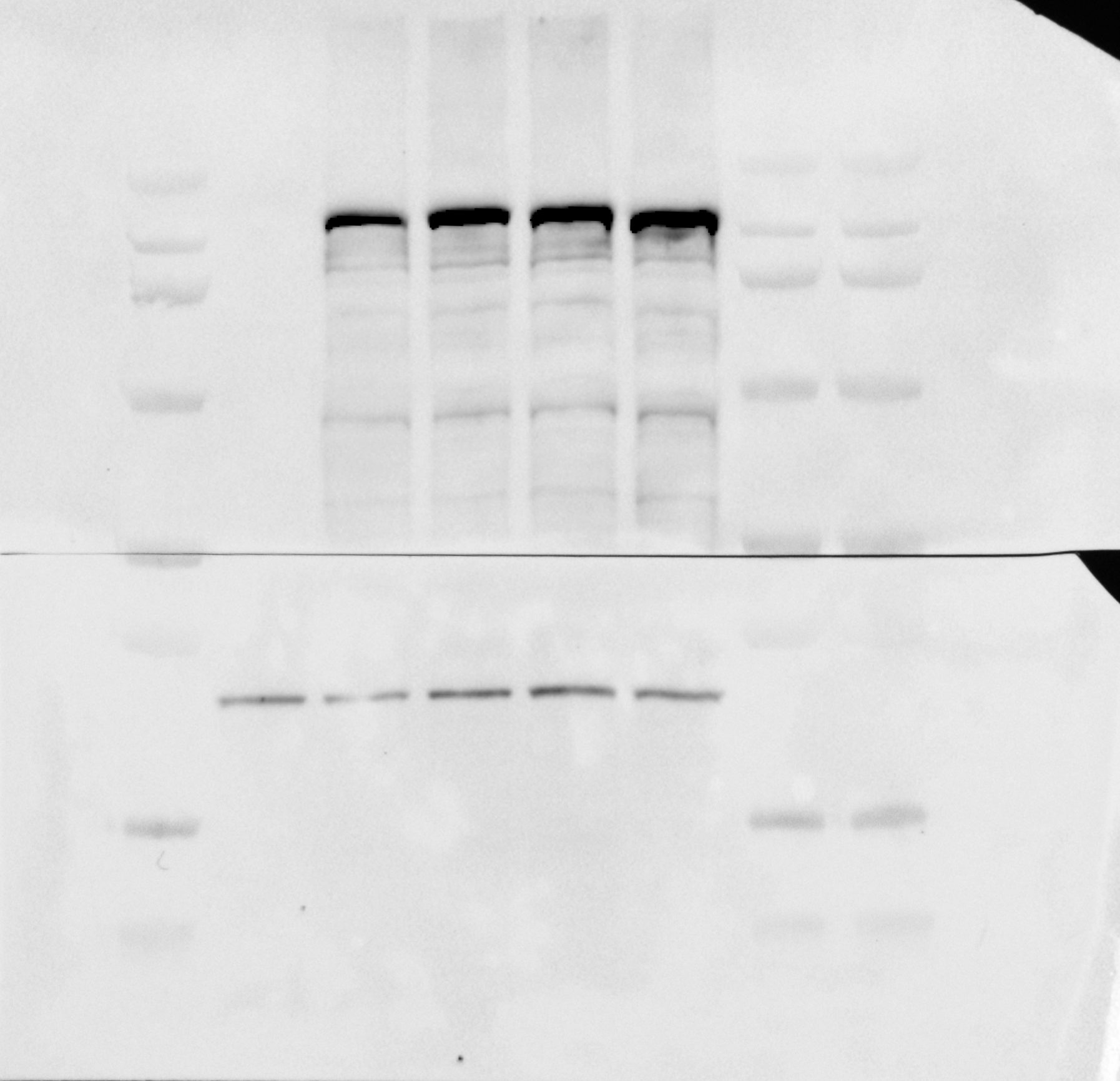

Supplement: Figure 3—figure supplement 1—source data 2. [file elife-102226-fig3-figsupp1-data2.zip › Figure 3-Figure Supplement 1-Source Data 2/Figure 3-Figure Supplement 1-Source Data 1-blot 1.tif]
